# Chirality-Induced Suppression of Singlet Oxygen in Lithium–Oxygen Batteries with Extended Cycle Life

**DOI:** 10.1007/s40820-025-01885-z

**Published:** 2025-08-25

**Authors:** Kyunghee Chae, Youngbi Kim, Yookyeong Oh, Hosik Hahn, Jaehyun Son, Youngsin Kim, Hyuk-Joon Kim, Hyun Jeong Lee, Dohyub Jang, Jooho Moon, Kisuk Kang, Jeong Woo Han, Filipe Marques Mota, Dong Ha Kim

**Affiliations:** 1 Department of Chemistry and Nanoscience, Ewha Womans University, 52 Ewhayeodae-Gil, Seodaemun-Gu, Seoul, 03760 Republic of Korea; 2 Department of Chemical Engineering, Pohang University of Science and Technology (POSTECH), Pohang, 37673 Republic of Korea; 3 Department of Materials Science and Engineering, Seoul National University, 1 Gwanak-Ro, Gwanak-Gu, Seoul, 08826 Republic of Korea; 4 Department of Materials Science and Engineering, Yonsei University, 50 Yonsei-Ro Seodaemun-Gu, Seoul, 03722 Republic of Korea; 5 Chemical and Biological Integrative Research Center, Korea Institute of Science and Technology (KIST), 5 Hwarang-Ro 14-Gil, Seongbuk-Gu, Seoul, 02792 Republic of Korea; 6 Department of Chemistry, School of Natural Sciences, University of Lincoln, Brayford Pool, Lincoln, LN6 7TS UK; 7 College of Medicine, Ewha Womans University, 25, Magokdong-Ro 2-Gil, Gangseo-Gu, Seoul, 07804 Republic of Korea; 8 Gradutate Program in Innovative Biomaterials Convergence, Ewha Womans University, 52 Ewhayeodae-Gil, Seodaemun-Gu, Seoul, 03760 Republic of Korea; 9 Basic Sciences Research Institute (Priority Research Institute), Ewha Womans University, Seoul, 03760 Republic of Korea; 10 Nanobio Energy Materials Center (National Research Facilities and Equipment Center), Ewha Womans University, Seoul, 03760 Republic of Korea

**Keywords:** Singlet oxygen suppression, Chirality-induced spin selectivity effect, Lithium–oxygen batteries, Oxygen evolution reaction, Battery stability

## Abstract

Lithium–oxygen (Li–O_2_) batteries are perceived as a promising breakthrough in sustainable electrochemical energy storage, utilizing ambient air as an energy source, eliminating the need for costly cathode materials, and offering the highest theoretical energy density (~ 3.5 kWh kg^–1^) among discussed candidates. Contributing to the poor cycle life of currently reported Li–O_2_ cells is singlet oxygen (^1^O_2_) formation, inducing parasitic reactions, degrading key components, and severely deteriorating cell performance. Here, we harness the chirality-induced spin selectivity effect of chiral cobalt oxide nanosheets (Co_3_O_4_ NSs) as cathode materials to suppress ^1^O_2_ in Li–O_2_ batteries for the first time. *Operando* photoluminescence spectroscopy reveals a 3.7-fold and 3.23-fold reduction in ^1^O_2_ during discharge and charge, respectively, compared to conventional carbon paper-based cells, consistent with differential electrochemical mass spectrometry results, which indicate a near-theoretical charge-to-O_2_ ratio (2.04 e^−^/O_2_). Density functional theory calculations demonstrate that chirality induces a peak shift near the Fermi level, enhancing Co 3*d*–O 2*p* hybridization, stabilizing reaction intermediates, and lowering activation barriers for Li_2_O_2_ formation and decomposition. These findings establish a new strategy for improving the stability and energy efficiency of sustainable Li–O_2_ batteries, abridging the current gap to commercialization.

**Supplementary Information:**

The online version contains supplementary material available at 10.1007/s40820-025-01885-z.

## Highlights

- Chiral cobalt oxide nanosheets (Co_3_O_4_ NSs) suppress singlet oxygen (^1^O_2_) generation in Li–O_2_ batteries via the CISS effect.
- *Operando* spectroscopy and density functional theory calculations confirm reduced parasitic reactions and enhanced oxygen electrochemistry.
- This strategy improves energy efficiency and cycle life, offering a path toward stable, high-performance Li–O_2_ batteries.

**Supplementary Information:**

The online version contains supplementary material available at 10.1007/s40820-025-01885-z.

## Graphical abstract

**Figure:**
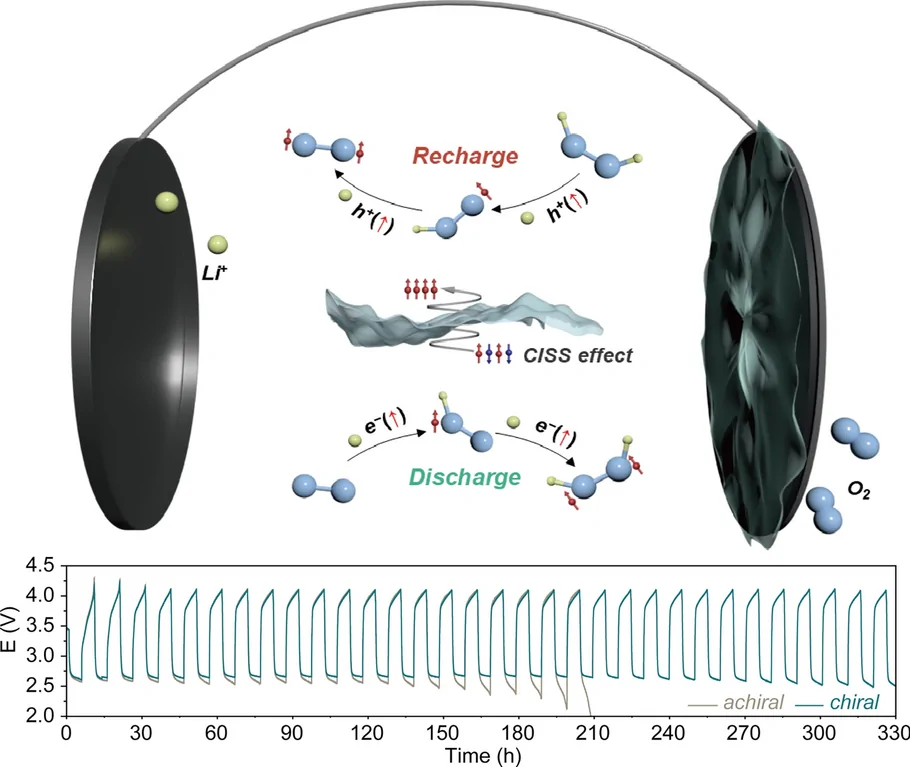

## Introduction

Aprotic lithium–oxygen (Li–O_2_) batteries are among the most chemically promising Generation After Next (GAN) systems for electrochemical energy storage, owing to their ultra-high theoretical energy density (~ 3,457 Wh kg^−1^), lightweight architecture—attributes shared with lithium–sulfur (Li–S) batteries [1–4]. Among the most sustainable energy storage technologies under development, Li–O_2_ and Li–S batteries stand out for their use of abundant elements [5–7]. However, the development of practical Li–O_2_ batteries faces significant challenges due to inherent instabilities in the oxygen electrochemistry, which drastically shortens the lifespan [8, 9]. In recent years, growing awareness of the detrimental effects of evolving singlet oxygen species (the ^1^∆_g_ excited state of oxygen, hereafter referred to as ^1^O_2_) has highlighted their contribution to side reactions that impair battery performance, reduce cyclability, and accelerate the degradation of key components [10]. Chemical oxidation of lithium peroxide (Li_2_O_2_), the primary discharge product in Li–O_2_ batteries, is a well-established source of singlet oxygen (^1^O_2_) generation. The formation of ^1^O_2_ in these systems becomes thermodynamically favorable at charging potentials exceeding 3.5–3.9 V vs. Li/Li^+^, based on the reversible potential of Li_2_O_2_ formation and the energy gap between triplet oxygen (^3^O_2_) and singlet oxygen (^1^O_2_) [11]. The highly reactive species promotes parasitic reactions during the battery recharge process, leading to the degradation of both the electrolyte and cathode materials [12]. Consequently, while Li–O_2_ batteries offer exceptional promise for long-duration energy storage and electrified aviation, overcoming these stability issues remains a fundamental barrier to realizing their long-term operation. Therefore, mitigating ^1^O_2_ generation and its consequent side reactions is a critical step toward advancing the development of reversible aprotic Li–O_2_ batteries.

To date, the suppression of ^1^O_2_ in aprotic Li–O_2_ batteries has primarily relied on ^1^O_2_ quenchers and redox mediators (RMs) [13–15]. Quenchers convert ^1^O_2_ to the more stable triplet oxygen (^3^O_2_), dissipating excess energy gently and mitigating harmful ^1^O_2_-related side reactions [12]. Nonetheless, the effectiveness of existing quenchers is limited by their narrow operational voltage window and insufficient quenching rate constants [12]. Alternatively, Liang et al. explored the dual role of several RMs in curbing ^1^O_2_ evolution while also serving as electron mediators, aiming to establish a correlation between the properties of RMs and their quenching capabilities [16]. However, recent findings indicate that RMs can decompose upon interaction with ^1^O_2_, resulting in a gradual decline in their catalytic effectiveness over repeated cycling [15]. Therefore, exploring alternative strategies for the effective suppression of ^1^O_2_ in these batteries is crucial. Another promising strategy to suppress ^1^O_2_ is leveraging the chirality-induced spin selectivity (CISS) effect, a unique property of chiral molecules. Due to their unique non-superimposable nature, these molecules facilitate spin-polarized currents through the CISS effect [17]. This phenomenon utilizes the helical potential of chiral molecules to align the spins of traversing electrons or holes, thereby reducing singlet oxygen formation, promoting triplet oxygen generation, and lowering oxygen evolution reaction (OER) overpotentials [18, 19].

In this study, we unveil for the first time the potential of the CISS effect in Li–O_2_ batteries by utilizing chiral cobalt oxide (Co_3_O_4_) as cathode catalysts. To ensure the sustainability of our strategy, we employed a simple electrodeposition method to design cost-effective low-loading Co_3_O_4_ nanosheets (NSs) with a high surface area able. Co_3_O_4_ has also been reported to offer stable performance and enhanced cyclability compared to alternative materials such as Pt and Ru [20–22]. Utilizing advanced real-time spectroscopic techniques, including *operando* differential electrochemical mass spectrometry (DEMS) to monitor the oxygen evolution during charging and *operando* photoluminescence (PL) to assess singlet oxygen suppression, we robustly demonstrate the impact of chirality on battery stability. Additionally, density functional theory (DFT) calculations were conducted to elucidate the discharge (DC) and recharge (RC) mechanisms, providing insight into the influence of spin orientation on reaction pathways. As a result, the modified Li–O_2_ batteries demonstrate significant improvements in cycle life and energy efficiency, outperforming conventional cell designs. This pioneering approach unveils the untapped potential of chiral materials for controlling reactive oxygen species within electrochemical energy storage systems. By strategically integrating chirality, this work establishes a new benchmark for achieving stability and performance in sustainable GAN energy storage technologies, paving the way for further advancements in the application of chiral materials in this field.

## Experimental Section

### Materials Preparation

Achiral and chiral cobalt oxide nanosheet (Co_3_O_4_ NSs) electrodeposition was carried out by a typical three-electrode system with the carbon paper (CP) substrates as the working electrode, a graphite rod utilized as a counter electrode, and a Ag/AgCl electrode serving as a reference electrode in a precursor solution consisting of 0.1 M Co(NO_3_)_2_∙7H_2_O in EtOH with 10 mM (*rac*/R/S)-1,1'-Bi-2-naphthol (BINOL) at the room temperature. The electrodeposition process was carried out through constant potential electrolysis at 1.2 V vs. Ag/AgCl for 10 min using potentiostat (Autolab electrochemical analyzer). As-prepared (*rac*/R/S)-Co_3_O_4_/CP electrode was rinsed with deionized water and EtOH and then dried at room temperature.

### Physical Characterization

The morphologies of the as-prepared sample were examined by high-resolution scanning electron microscopy (HR-SEM) on JSM-7610F systems. Powder X-ray diffraction (XRD) patterns were collected under Ni filtered Cu-Kα radiation (λ = 1.5418 Å) in the 10° to 70° 2θ region without air exposure using a Rigaku Dmax 2000 diffractometer. X-ray photoelectron spectroscopy (XPS) was performed on a Thermo Scientific K-Alpha XPS, using a dual-beam source and ultra-low energy electron beam.

The circular dichroism (CD) spectra were recorded on a JASCO J-1500 CD spectrometer. In addition, mc-AFM (SPA400, Seiko Instruments) was performed in an area of 2 μm × 2 μm using a Co-Cr-coated cantilever to investigate the electrical properties of the devices. Prior to the measurement, tips were premagnetized by a strong permanent magnet for > 60 min. For all samples, *I-V* curves were measured by sweeping the voltage from -3 to + 3 V with a frequency of 0.5 Hz. In each of the mc-AFM measurements, the average value was obtained from 30 times of *I-V* sweeps with different positions and samples.

### Li–O_2_ Cell Assembly

Li foil (Honjo) was used as the anode in all assembled swagelok-type batteries. Li foil was punched as disk (*d* = 1.27 cm) and pressed on to stainless-steel current collectors of the same size. Pieces of Celgard® H2010 20 μm microporous trilayer membrane (*d* = 2 cm) and glass microfiber separators (Whatman, GF/C, *d* = 1.5 cm) were dried under vacuum for at least 48 h at 80 °C and under vacuum 110 °C, respectively. Commercial tetraethylene glycol dimethyl ether (TEGDME, Panax. Etec. Co.) with 1.0 M lithium bis(trifluoromethanesulfonyl)imide (LiTFSI) was used as the electrolyte in all experiments here carried out. The punched Li foil, the Celgard film, the glass microfiber separator, and the cathode were sequentially assembled inside the Ar-filled glove box (Mbraun) under < 0.5 ppm H_2_O and < 0.5 ppm O_2_. The prepared electrolytes (total of 120 μL) were added in between the components. The cells were then purged with O_2_ (99.995%) at atmospheric pressure for 2 min and cycled at a constant temperature (26 °C) inside an incubator after 1 h-rest period at open circuit, using a WonATech WBCS 3000 multi-channel battery testing system.

### *Operando* PL Analysis

Fluorescence measurements were recorded with a spectrofluorometer (JASCO, FP-8500) with a 150 W Xe lamp for the excitation light source. The *operando* fluorescence measurements were performed in the front face mode in kinetic acquisition mode with 0.1 s excitation every 10 s to minimize photobleaching of the DMA. The electrolyte was 0.1 M LiTFSI in TEGDME containing 20 mM DMA as a ^1^O_2_ trap. DMA was excited at 378 nm, and the emission was detected at 425 nm. The homemade *operando* PL cell was constructed with a customized gas-tight quartz window, sealed with a rubber septum to prevent ambient contamination. High-purity O_2_ gas (99.995%) was introduced into the cell via a precision-controlled gas inlet, ensuring complete electrolyte saturation before measurement. The purging process was conducted for 10 min at a controlled flow rate to ensure reproducibility. This specialized design enabled stable and accurate operando monitoring of singlet oxygen evolution. The cell consisted of a sealed chamber with an integrated electrode holder, allowing precise alignment of the working electrode and counter electrode. Li foil (anode) and as-prepared chiral electrode (cathode) were punched as 1 × 1 cm^2^ and secured within the setup. The assembly was performed in an Ar-filled glovebox and then purged with O_2_ for 10 min at atmospheric pressure before cycling at a constant temperature (26 °C) inside the fluorescence spectrometer using an electrochemical workstation (SP150).

### *Operando* DEMS Analysis

Differential electrochemical mass spectroscopy (DEMS) was performed to analyze the gases evolving from the Li–O_2_ cell during charging. The homemade *operando* DEMS system was composed of a mass spectrometer (MS) (HPR-20, Hiden Analytical, UK) and a potentio-galvanostat. For DEMS analysis, the Li–O_2_ cells were initially discharged. Then, the cells were connected to the MS to detect the volume of gases evolved during the charging process. Before the charging process, the cells were fully rested in Ar environments for 4 h. The MS was calibrated for O_2_, using an Ar-based mixture gas.

### DFT Calculations

DFT calculations were conducted using the Vienna ab initio simulation package and employed the projector augmented wave (PAW) method with spin polarization [23, 24]. The Perdew–Burke–Ernzerhof functional of the generalized gradient approximation was used for the exchange–correlation energies [25–27]. In all calculations, van der Waals interactions were considered by the Grimme DFT-D3 method. The kinetic cutoff energy was set to 415 eV, and a Brillouin zone was sampled using 1 × 1 × 1 Gamma k-point grids [28, 29]. The convergence criterion of the energy was 1 × 10^–6^ eV for the self-consistent-field iterations. Based on a force-based conjugated gradient algorithm, all the calculations were performed until the forces on each atom were within 0.03 eV Å^−1^. The DFT + U method was used to treat the strong onsite coulomb interactions [30]. An effective U value of 3.0 eV was chosen to correctly describe Co [31]. The following equation was used to acquire the free energy: G = E + ZPE-TS, where G is free energy, E is total energy calculated by DFT, ZPE is the zero-point energy, and TS is the entropy contribution (T is set as 298.15 K) [32]. The adsorption energies (E_ads_) were calculated based on the formula E_ads_ = E_total_-E_slab_-E_ads_, where E_slab_ and E_ads_ are the total energies of the optimized total system, the clean slab, and the adsorbate in the structure, respectively.

## Results and Discussion

### Fabrication and Characterization of Electrode

**Fig. 1:**
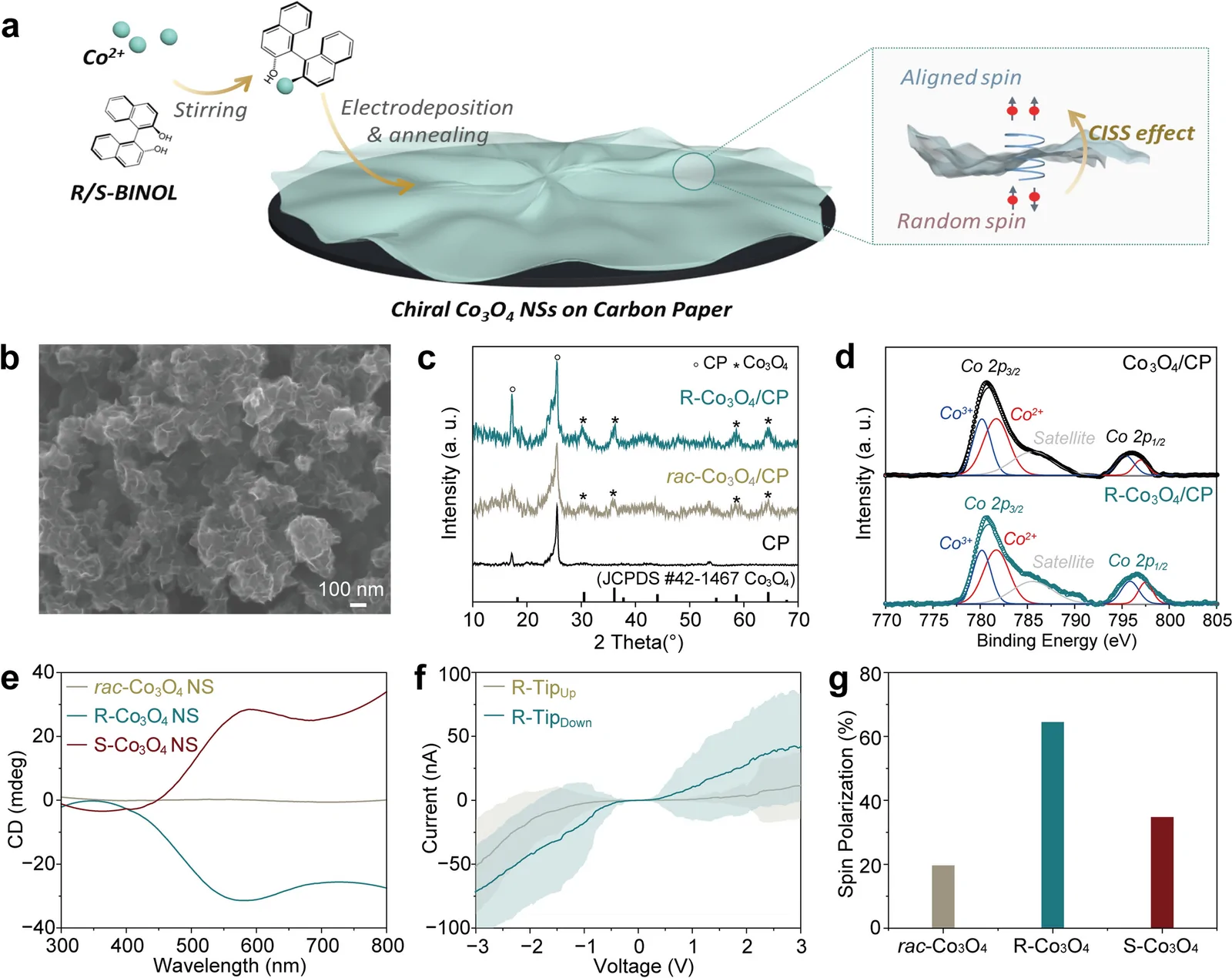
Characterizations of chiral Co_3_O_4_ NS/CP.** a** Schematic illustration of the fabrication of chiral Co_3_O_4_ NS on CP. **b** SEM image of R-Co_3_O_4_ NS/CP. **c** XRD patterns of CP, Co_3_O_4_ NS/CP, and R-Co_3_O_4_ NS/CP. **d** XPS spectra of Co_3_O_4_ NS/CP and R-Co_3_O_4_ NS/CP. **e** CD spectra of (*rac*/R/S)-Co_3_O_4_ NS on ITO substrates. **f** *I-V* curves of R-Co_3_O_4_ NS/CP with spin polarization percentage (SP%) in the range of –1.5 to + 1.5 V. The CoCr tip was magnetized along the north (gray) or south (blue) orientation. The average *I-V* curve recorded over 30 scans at different points is shown. **g** Spin polarization percentage (SP%) as a function of applied bias of R-Co_3_O_4_/CP

Chiral Co_3_O_4_ nanosheets were electrodeposited onto carbon paper (CP) from an ethanol electrolyte solution containing a Co (II) precursor and (R)-1,1′-Bi-2-naphthol (R-BINOL) as a chirality inducer (Figs. 1a and S1). Scanning electron microscopy (SEM) demonstrated the uniform growth of these nanosheets on the CP framework (Figs. 1b and S2), establishing a free-standing binder-free structure with a low-resistance pathway for electron transfer [33, 34]. Dynamic light scattering (DLS) measurements (Fig. S3) revealed that the lateral sizes of *rac*-Co_3_O_4_ NS, R-Co_3_O_4_ NS, and S-Co_3_O_4_ NS were 324, 360, and 335 nm, respectively. In addition, atomic force microscopy (AFM) images (Fig. S4) revealed a nanosheet-coated electrode surface with a uniform thickness of 140–160 nm, indicating consistent deposition of the Co_3_O_4_ nanosheets-layer across the ITO substrates. X-ray diffraction (XRD) was used to investigate the crystal structures of both the achiral and chiral samples, referred from hereon as *rac*-Co_3_O_4_/CP and R-Co_3_O_4_/CP, respectively (Fig. 1c). The XRD patterns for both electrodes were in good agreement with established data for spinel cobalt oxide [35]. X-ray photoelectron spectroscopy (XPS) was employed to analyze the chemical states of the synthesized electrodes, as depicted in Figs. 1d and S5. Peak fitting analysis of the Co 2*p* spectra from both the bare Co_3_O_4_/CP (without the ligand) and the chiral R-Co_3_O_4_/CP identified two chemical states, Co 2*p*_3/2_ and Co 2*p*_1/2_, with no noticeable shifts [36, 37]. Deconvolution of the Co 2*p*_3/2_ region revealed consistent Co^2+^/Co^3+^ ratios of 1.44 (bare Co_3_O_4_/CP), 1.46 (*rac*-Co_3_O_4_/CP), and 1.43 (R-Co_3_O_4_/CP), respectively. This confirms that inducing chirality does not fundamentally alter the cobalt oxidation state, in agreement with previous studies on spinel-type Co_3_O_4_ materials [35–37]. Furthermore, to evaluate the cobalt content in the electrodes, inductively coupled plasma optical emission spectroscopy (ICP-OES) was performed (Table S1). The results revealed a low cobalt loading across all samples, with 5.01 wt% for R-Co_3_O_4_/CP, confirming minimal catalyst content and enabling fair electrochemical comparison.

The circular dichroism (CD) spectra of these electrodes, synthesized from R-BINOL:Co (II) (R-Co_3_O_4_ NSs) and S-BINOL:Co (II) (S-Co_3_O_4_ NSs), are displayed in Figs. 1e and S6. These spectra demonstrate an approximate mirror symmetry, illustrating the opposite chirality of the Co_3_O_4_ NSs grown from solutions containing different Co-BINOL chirality [38]. Conversely, electrodeposition using *rac*-BINOL:Co (II) complexes, which do not exhibit net chirality due to the inclusion of both chiral centers in *rac*-BINOL, produced an achiral film. The spin-dependent currents of the chiral Co_3_O_4_/CP were investigated using magnetic conductive probe atomic force microscopy (mc-AFM), as shown in Fig. S7. Utilizing ferromagnetic CoCr tips premagnetized northward and southward allowed for the measurement of spin-polarized currents within a voltage range of –3.0 to 3.0 V. For consistent results, more than 30 distinct points on each sample were examined. Notably, currents under the Tip_Down_ condition for the R-Co_3_O_4_/CP significantly surpassed those in the Tip_Up_ configuration, suggesting a preference for down-spin charge carriers (Fig. 1f). Conversely, the S-Co_3_O_4_/CP exhibited increased currents with upward tip magnetization (Fig. S7c). No notable changes in current were detected in the achiral *rac*-Co_3_O_4_/CP under different tip magnetizations (Fig. S5a). The spin polarization (SP) of the chiral Co_3_O_4_/CP was calculated using the [Eq. (1)]:

1 $$SP\left( \% \right) = \left( {I_{Up} - I_{Down} } \right)/\left( {I_{Up} + I_{Down} } \right) \times 100\%$$

where *I*_*Up*_ and *I*_*Down*_ represent the currents measured with the CoCr tip magnetized northward and southward, respectively [39]. Figure 1g highlights the distinct spin-dependent current behavior of the R-Co_3_O_4_/CP, which exhibited an average spin polarization (SP) of 64.4%. In contrast, the *rac*-Co_3_O_4_/CP showed no difference between *I*_*Up*_ and *I*_*Down*_ currents, indicating that the spin-dependent behavior is linked to the induced chirality in Co_3_O_4_, a direct outcome of the CISS effect [40].

### Battery Performance with and without Chirality

**Fig. 2:**
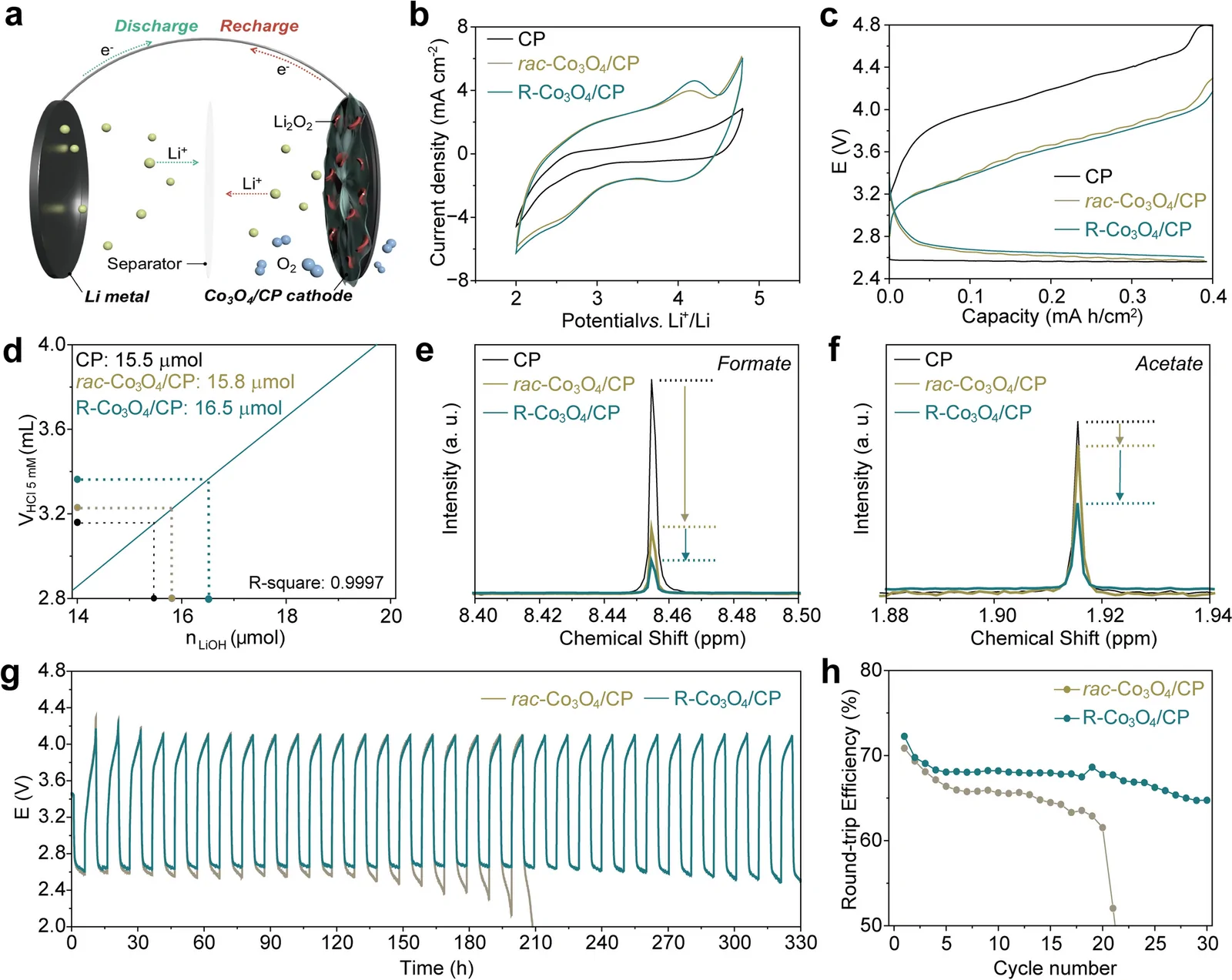
Battery performance of chiral Co_3_O_4_ NS/CP.** a** Schematic illustration of the fabrication of Li–O_2_ battery. **b** Cathodic CV curves of the Li–O_2_ batteries with CP, *rac*-Co_3_O_4_ NS/CP, and R-Co_3_O_4_ NS/CP at a scan rate of 0.1 mV s^–1^ between 2.0 and 4.8 V. **c** Galvanostatic cycling of Li–O_2_ batteries with CP, *rac*-Co_3_O_4_ NS/CP, and R-Co_3_O_4_ NS/CP at 0.08 mA cm^−2^. **d** Calculated Li_2_O_2_ amount using UV–Vis titration experiments. **e**, **f** ^1^H NMR spectra for collected CP, *rac*-Co_3_O_4_ NS/CP, and R-Co_3_O_4_ NS/CP cathodes after DC for 5 h in (**e**) Formate region and (**f**) Acetate region. **g** Cycling performance of the Li–O_2_ batteries with *rac*-Co_3_O_4_ NS/CP and R-Co_3_O_4_ NS/CP cathodes under galvanostatic conditions at 0.08 mA cm^–2^, using a fixed charge/discharge time of 10 h per cycle, corresponding to a capacity cutoff of 0.4 mAh cm^–2^. **h** Round-trip efficiencies versus cycle number. Cells were cycled at a fixed capacity of 0.4 mAh cm^–2^ and a constant current of 0.08 mA cm^–^.^2^

Prepared electrodes were assessed for their charging potential, catalytic efficiency, and cyclability in Li–O_2_ cells (Fig. 2a). The oxygen electrochemical reactions within these cells were first examined using cyclic voltammetry (CV). Figure 2b illustrates the typical CV response in the Li–O_2_ cell at a scan rate of 0.1 mV s^–1^, spanning from 2.0 to 5.0 V vs Li/Li^+^. The R-Co_3_O_4_/CP cathode demonstrated higher oxygen reduction reaction (ORR) and oxygen evolution reaction (OER) current densities compared to the bare CP. In good agreement, both LSV curves obtained with (R/S)-Co_3_O_4_/CP exhibited enhanced catalytic activity against the achiral and CP references (Fig. S8). Notably, the ORR performance of R-Co_3_O_4_/CP and S-Co_3_O_4_/CP is largely comparable, showing minimal differences in onset potential and current density (Fig. S8b). This is consistent with the fact that ORR in aprotic Li–O_2_ systems primarily involves an outer-sphere electron transfer to O_2_, a spin-conserved process that does not significantly benefit from spin polarization [41]. In contrast, OER processes involve bond rearrangements and intermediate states where spin selection can play a role. These assumptions agree, therefore, with the more pronounced enhancement in R-Co_3_O_4_/CP compared to S-Co_3_O_4_/CP [41]. The higher spin polarization of R-Co_3_O_4_/CP, as confirmed by mc-AFM (Fig. 1g), likely contributes to its superior OER performance. These findings highlight enhanced electrochemical performance in both the formation and decomposition of discharge products.

The first discharge/charge profiles of Li–O_2_ cells in an O_2_-saturated electrolyte (1.0 M LiTFSI in G4) at 0.08 mA cm^–2^ are presented in Fig. 2c. Both discharge and, notably, charge overpotentials show improvement with Co_3_O_4_ NSs, in good agreement with the reported catalytic properties and the previous literature [35, 42]. Interestingly, however, negligeable differences in overpotential were observed between *rac*-Co_3_O_4_/CP and R-Co_3_O_4_/CP during the first cycle. As discussed in the following sections and well established in the literature, these results agree well with the impact of singlet oxygen being more pronounced throughout cyclability [43–45].

To have a better understanding of the battery performance, we then analyzed the nature of the formed discharge products using titration and ^1^H NMR methods. Acid–base titrations of spent cathodes, using phenolphthalein as an indicator, were conducted to estimate the formation of lithium peroxide (Li_2_O_2_) against theoretical predictions. The discharged R-Co_3_O_4_/CP cathodes delivered an 87% Li_2_O_2_ yield (Figs. 2d and S9 for details), with both *rac*-Co_3_O_4_/CP and the bare CP attaining lower yields (84 and 82%, respectively), suggestive of the role chirality plays in mitigating side reactions and enhancing product formation during discharge. We then compared ^1^H NMR results assessing the formation of carboxylate side products, which reflect the reactivity of ^1^O_2_ during cycling. During discharge, ^1^O_2_ reacts with the organic solvents in the electrolyte, leading to oxidative cleavage of C-H bonds or C-C bonds in these organic molecules. This reaction produces reactive intermediates like peroxide, radicals (e.g., R^•^, ROO^•^), which further degrade into small organic acid, including formate and acetate species [46, 47]. The formation of lithium formate and lithium acetate side products was, therefore, surveyed through the detection of HCOOD and CH_3_COOD peaks in the NMR solvent [12]. Summarized results evidenced a striking suppression of formate species in Co_3_O_4_ NSs-containing cells (Fig. 2e). Most importantly, the introduction of chirality with R-Co_3_O_4_/CP further reduced the generation of lithium formate, while remarkably hindering emerging acetate side products (Fig. 2f). Taken together, these results suggest that the CISS effect can mitigate the generation of singlet oxygen through the introduction of chirality, reducing the formation of resulting side products. This understanding highlights the potential of chiral materials in enhancing the stability and efficiency of Li–O_2_ cells by suppressing detrimental side reactions.

Having shed light on the nature of species formed during the first cell cycle, we then assessed the cyclability of cells with and without induced chirality and their correlation with the accumulation of side products [10]. As depicted in Fig. S10, R-Co_3_O_4_/CP displayed remarkable stability, with minimal potential shifts across the 1^st^, 2^nd^, and 5^th^ charge profiles. Notably, the voltages required during recharge steps were consistently lower by approximately 0.4–0.5 V compared to the bare CP electrode, demonstrating the catalytic advantage of the chiral material in reducing charge overpotentials. As illustrated in Fig. 2g, the cell assembled with *rac*-Co_3_O_4_/CP, operating at 0.08 mA cm^–2^ with a capacity cutoff of 0.4 mAh cm^–2^, sustained only 20 cycles before capacity fading, reaching ca. 200 h of operation. In contrast, the R-Co_3_O_4_/CP-containing cell demonstrated stable performance beyond 300 h (33 cycles), with no noticeable potential shifts during discharge/recharge cycles. Corresponding round-trip efficiencies (Fig. 2h) further corroborated the beneficial impact of chirality, revealing superior energy utilization over extended cycling. To verify the retention of catalytic activity after extended cycling, CVs were collected on the CP, *rac*-Co_3_O_4_/CP, and R-Co_3_O_4_/CP electrodes following 10 discharge–recharge galvanostatic cycles. As shown in Fig. S11, whereas all three electrodes exhibited a decrease in current density, the R-Co_3_O_4_/CP electrode retained the highest ORR and OER activity. In contrast, rac-Co_3_O_4_/CP and CP electrodes showed more pronounced declines, particularly in the OER region. These results highlight the superior structural integrity and catalytic durability of the chiral electrode under repeated cycling conditions.

### *Operando* Detection of Singlet Oxygen

Figure S12 schematically illustrates the chemical and electrochemical routes leading to the generation of ^1^O_2_ in Li–O_2_ battery [43]. During DC, molecular oxygen (O_2_) undergoes a one-electron reduction to form superoxide anions (O_2_^•–^) [Eq. (2)], which can either be further reduced and react with Li^+^ to form lithium peroxide (Li_2_O_2_) [Eq. (3)] or undergo disproportionation to yield both ^1^O_2_ and ^3^O_2_ along with Li_2_O_2_ formation [Eq. (4)] depending on solvation and surface interactions [43].

2 $$O_{2} \left( g \right) + e^{ - } \to O_{2}^{ \bullet - }$$

3 $$O_{2}^{ \bullet - } + e^{ - } + 2Li^{ + } \to Li_{2} O_{2} \left( s \right)^{*}$$

4 $$2O_{2}^{ \bullet - } + 2Li^{ + } \to Li_{2} O_{2} \left( s \right) + ^{3} O_{2} /^{1} O_{2}$$

During RC, Li_2_O_2_ decomposes via multiple reaction channels. Initially, delithiation takes place at the Li_2_O_2_ lattice [Eq. (5)], forming a Li-deficient oxide intermediate (Li_2-x_O_2_). The latter then decomposes to either ^1^O_2_/^3^O_2_ [Eq. (6)] or releases soluble superoxide species that undergo further oxidation [Eq. (7)]. Both pathways contribute to ^1^O_2_ evolution, depending on the O_2_^•–^ solvation properties of the electrolyte [43].

5 $$Li_{2} O_{2} \left( s \right) \to Li_{2 - x} O_{2} \left( s \right) + xe^{ - } + xLi^{ + }$$

6 $$Li_{2 - x} O_{2} \left( s \right) \to ^{3}O_{2} /^{1}O_{2} + \left( {2 - x} \right)e^{ - } + \left( {2 - x} \right)Li^{ + }$$

7 $$Li_{2 - x} O_{2} \left( s \right) \to O_{2}^{ \bullet - } \left( {sol} \right) + \left( {1 - x} \right)e^{ - } + \left( {2 - x} \right)Li^{ + } \to ^{1} O_{2}$$

These reactions demonstrate that singlet oxygen generation occurs via both surface-mediated disproportionation and direct electrochemical oxidation of superoxide intermediates. During the DC step, disproportionation of O_2_^•–^ can yield ^1^O_2_, although this is less favorable due to the thermodynamic stability of ^3^O_2_. In contrast, during RC, the oxidation of Li_2_O_2_ produces O_2_^•–^ intermediates, which can undergo further oxidation to ^1^O_2_ as the cell voltage exceeds 3.55 V [48]. The extent of ^1^O_2_ generation is influenced by the solvation ability of O_2_^•–^ in the electrolyte and the reversibility of Li_2_O_2_ decomposition, highlighting the critical importance of controlling superoxide pathways in improving Li–O_2_ battery stability.

**Fig. 3:**
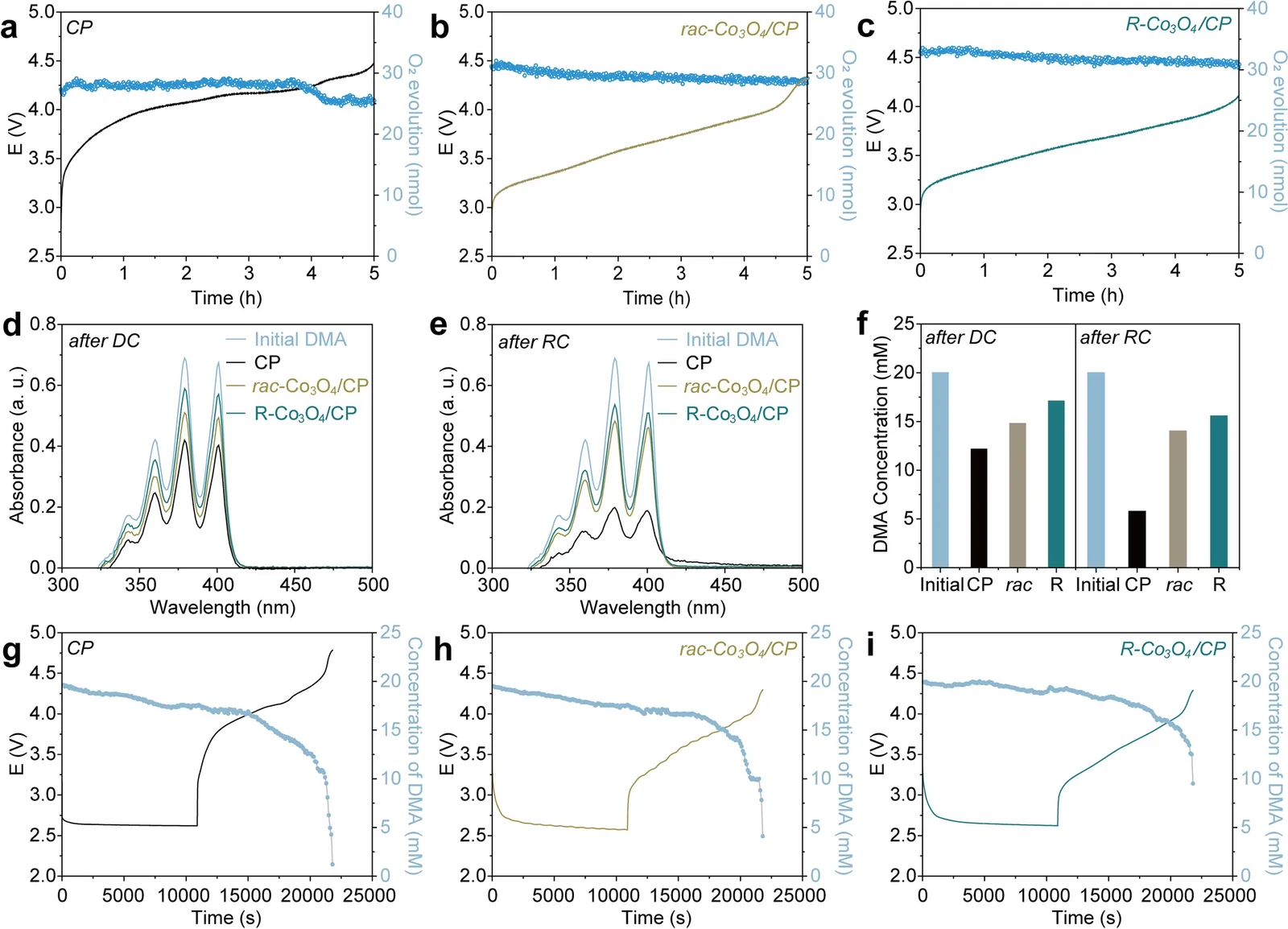
Detection of singlet oxygen. **a-c** Quantification of the product and by-products during charge using *operando* DEMS with (**a**) CP, (**b**) *rac*-Co_3_O_4_ NS/CP, and (**c**) R-Co_3_O_4_ NS/CP electrode during charge at 0.08 mA cm^–2^ for 5 h. **d**, **e** UV–Vis absorption spectrum of DMA in G4 with CP, *rac*-Co_3_O_4_ NS/CP, and R-Co_3_O_4_ NS/CP after (**d**) discharge and (**e**) recharge for 1 h at 0.08 mA cm^–2^. **f** Calculated DMA concentration after DC and RC with CP, *rac*-Co_3_O_4_ NS/CP, and R-Co_3_O_4_ NS/CP. **g-i** *Operando* PL spectroscopy characterizing DMA consumption during charge with (**g**) CP, (**h**) *rac*-Co_3_O_4_ NS/CP, and (**i**) R-Co_3_O_4_ NS/CP electrode during galvanostatic discharge and charge at 0.08 mA cm.^–2^

Mechanistic insight was first offered using *operando* differential electrochemical mass spectrometry (DEMS) of cells operated at 0.08 mA cm^–2^ for 5 h. Evolved O_2_ gases were recorded throughout the recharge step of the assembled cells. In Fig. 3a, the charge voltage for the reference Li–O_2_ battery with CP reaches up to 4.2 V, accelerating ^1^O_2_ generation and leading to significant side reactions [16]. As a result, a high charge-to-O_2_ ratio (2.34 e^–^/O_2_) is observed, reflecting poor stability and inefficiency in O_2_ evolution. For the Li–O_2_ battery with *rac*-Co_3_O_4_/CP (Fig. 3b), the system shows better reversibility with a charge-to-O_2_ ratio of 2.13 e^–^/O_2_. Comparatively, the Li–O_2_ battery with R-Co_3_O_4_/CP exhibits an almost exclusive evolution of O_2_, with a charge-to-O_2_ ratio close to the theoretical value (2.04 e^–^/O_2_), indicating high round-trip reversibility and a nearly stoichiometric evolution of the molecular O_2_ consumed during the preceding discharge step (Fig. 3c).

To establish a correlation between the performance enhancement above uncovered and the expected prevention of ^1^O_2_ formation and the related parasitic reactions, we then conducted UV–vis spectroscopy using 9,10-dimethylanthracene (DMA) as a well-established probe to quantify ^1^O_2_ generation. The selective reaction of DMA with ^1^O_2_ forms endoperoxide (DMA-O_2_), with a resulting decline in the DMA characteristic absorbance peak at 379 nm [12]. The change in absorbance is directly proportional to the amount of ^1^O_2_ generated. Li–O_2_ batteries with CP, *rac*-Co_3_O_4_/CP, and R-Co_3_O_4_/CP cathodes were assembled in a reference electrolyte containing 20 mM DMA. Figure 3d, e summarizes the results following 1 h discharge and recharge periods at 0.08 mA cm^−2^. During DC, quantitative analysis of DMA consumption was strikingly suppressed with the R-Co_3_O_4_/CP cathode (10.5%) compared to the *rac*-Co_3_O_4_/CP (26.1%) and CP counterparts (39.2%) (Figs. 3d and S13a). During RC, a similar trend was observed, with the R-Co_3_O_4_/CP cathode showing significantly reduced DMA consumption (22%) compared to *rac*-Co_3_O_4_/CP (30%) and CP (71.1%) (Figs. 3e and S13b). Taken together, the R-Co_3_O_4_/CP cathode exhibited a 3.7-fold lower consumption than the CP reference during the discharge step and a 3.23-fold reduction during the recharge step, respectively, underscoring its superior ability to suppress ^1^O_2_ generation (Fig. 3f). This behavior is consistent with superior evolution of molecular oxygen in the presence of R-Co_3_O_4_/CP, uncovered during DEMS measurements (Fig. 3c) [8, 12, 49].

To further corroborate the conclusions drawn above, an *operando* fluorescence (PL) setup, conveniently schematized in Fig. S14, was developed to monitor real-time changes during cell operation. The setup, pairing a 1 × 1 cm^2^ Li foil with a 1 × 1 cm^2^ working electrode in a larger electrolyte volume of 2 mL, included a gas-tight quartz cuvette with a slightly pressurized O_2_ headspace. CP, *rac*-Co_3_O_4_/CP, and R-Co_3_O_4_/CP working electrodes were immersed in the O_2_-saturated reference electrolyte containing 20 mM DMA. Excitation and emission wavelengths were chosen based on DMA’s characteristic peaks (Fig. S15) for optimal sensitivity in tracking ^1^O_2_. Figure 3g-i shows the voltage profile and DMA concentration during galvanostatic discharge and charge at 0.08 mA cm^–2^. During the 3-h discharge step, DMA consumption followed the trend R-Co_3_O_4_/CP (5% DMA consumption) < *rac*-Co_3_O_4_/CP (12.5%) < CP (14%), highlighting the enhanced suppression of ^1^O_2_ generation by the chiral electrode. In line with the results above, a more pronounced decrease in DMA concentration and corresponding PL intensity was witnessed during charge, particularly at voltages exceeding 3.55 V. Nonetheless, the R-Co_3_O_4_/CP cathode (Fig. 3i) exhibited significantly lower DMA consumption (52.5%) compared to *rac*-Co_3_O_4_/CP (79.5%, Fig. 3h) and CP (94%, Fig. 3g). These results consistently demonstrate the superior ability of the chiral electrode to suppress ^1^O_2_ formation during both discharge and recharge processes.

### Reaction Mechanism

**Fig. 4:**
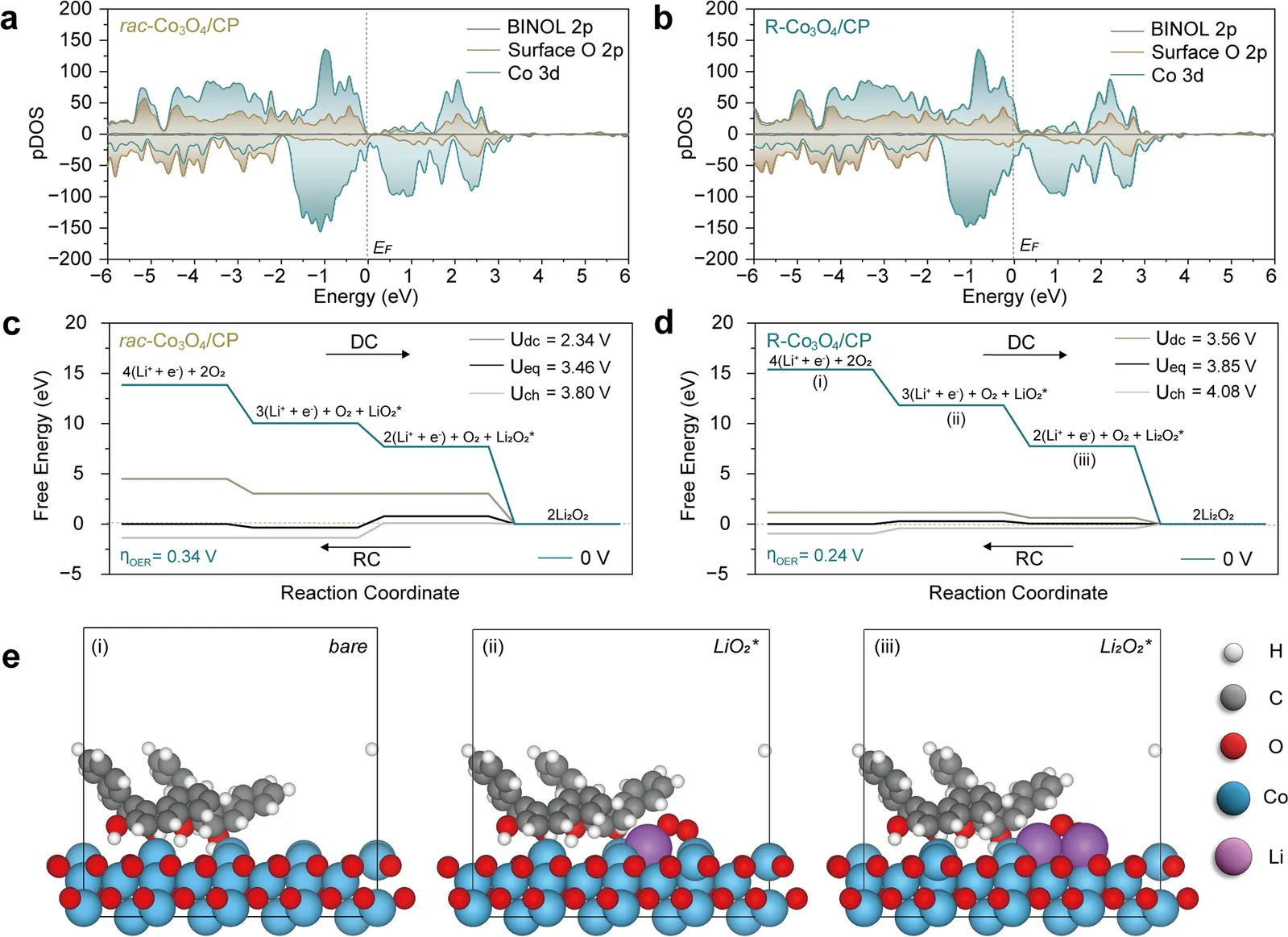
Reaction mechanism.** a**, **b** Total density of states (pDOS) of (**a**) *rac*-Co_3_O_4_ NS/CP and (**b**) R-Co_3_O_4_ NS/CP electrode. **c**, **d** The Gibbs free energy profiles of ORR/OER process on the (**c**) *rac*-Co_3_O_4_ NS/CP and (**d**) R-Co_3_O_4_ NS/CP cathodes. **e** The computed geometries of (**i**) bare, (**ii**) LiO_2_*, and (**iii**) Li_2_O_2_* at the R-Co_3_O_4_ NS/CP cathode

To shed light on the reaction mechanism, we then conducted projected density of states (pDOS) calculations (Fig. 4a, b). Results first reveal a shift in the electronic structure of the R-Co_3_O_4_/CP system against its racemic counterpart. In detail, a clear peak shift near the Fermi level (*E*_*F*_) is observed with the R-Co_3_O_4_/CP system, indicating an increased projected density of states near *E*_*F*_, particularly from Co 3*d* and O 2*p* orbitals, which favors charge transfer efficiency and facilitating improved electronic conductivity [50]. This is in strong agreement with the observed enhancement in ORR/OER activity in the CV and LSV measurements (Figs. 2b and S8), confirming improved charge transport characteristics. The increased pDOS near *E*_*F*_ allows more available states for electron transfer, particularly during Li_2_O_2_ formation (DC) and decomposition (RC), enhancing the rate of electron injection or withdrawal from surface-bound intermediates such as LiO_2_*. This is accompanied by a strong orbital alignment between Co 3*d* and O 2*p* states, with extended hybridization between metal and oxygen atoms (Figs. S16 and S17). Given the critical role of σ-bonding interactions between transition metal 3*d* and oxygen 2*p* orbitals in O_2_ electrochemistry occurring during discharge/recharge steps, this alignment enhances electron transport, with optimized reaction kinetics during cycling [51]. As a result, the stronger hybridization stabilizes oxygenated intermediates, lowers activation barriers for O–O bond formation and cleavage, and accelerates overall redox kinetics. These features are consistent with the reduced overpotential and improved round-trip efficiency observed in R-Co_3_O_4_/CP electrode cells.

Gibbs free energy calculations (Fig. 4c, d) further corroborate the improved catalytic performance of R-Co_3_O_4_/CP, with theoretically lower overpotentials (η_ORR_ = 0.29 V, η_OER_ = 0.24 V) against the *rac*-Co_3_O_4_/CP (η_ORR_ = 1.12 V, η_OER_ = 0.34 V). These results are in good agreement with the introduction of chirality modulating spin-dependent electronic interactions and leading to selective spin transport that stabilizes key reaction intermediates. This stabilization reduces activation barriers for both ORR and OER, accelerating electron transfer dynamics at the electrode–electrolyte interface, and enabling a more efficient cycling process. The free energy profile suggests that the initial LiO_2_ formation step (Fig. 4d) requires a lower activation energy, leading to faster Li_2_O_2_ accumulation during discharge. This aligns with experimental titration results (Fig. 2d), where R-Co_3_O_4_/CP demonstrated a higher Li_2_O_2_ yield compared to *rac*-Co_3_O_4_/CP, confirming a more efficient discharge mechanism. The strong Co 3*d*–O 2*p* hybridization in the R-Co_3_O_4_/CP system further reinforces this effect, by facilitating rapid charge transfer. This improvement in charge transport is directly reflected in the suppression of parasitic reactions, as demonstrated by the significant reduction in formate and acetate species detected in ^1^H NMR (Fig. 2e, f). Conversely, upon recharge, the decomposition of Li_2_O_2_ is more energetically favorable on R-Co_3_O_4_/CP, which explains the improved cycling stability observed experimentally (Fig. 2g), as well as the reduced singlet oxygen generation evidenced by DMA consumption (Fig. 3e, f).

Adsorption energy calculations (Figs. 4e and S18) also reveal that the R-Co_3_O_4_/CP system favors a surface-mediated Li_2_O_2_ growth mechanism, in which LiO_2_ adsorption is weakened, while Li_2_O_2_ stabilization is strengthened [52]. This theoretical finding correlates with the DEMS results (Fig. 3c), where R-Co_3_O_4_/CP exhibited a nearly stoichiometric charge-to-O_2_ ratio, indicating improved oxygen evolution efficiency and enhanced Li_2_O_2_ decomposition during recharge. This prevents the accumulation of reactive LiO_2_ intermediates known to facilitate singlet oxygen generation and induce subsequent parasitic side reactions. The charge density difference plots further highlight the stronger electronic interaction between Li_2_O_2_ and the R-Co_3_O_4_ surface compared to *rac*-Co_3_O_4_ (Fig. S19), which contributes to the improved discharge and recharge efficiency. By promoting a surface-controlled Li_2_O_2_ formation pathway, the R-Co_3_O_4_/CP system effectively mitigates ^1^O_2_ generation and enhances the reversibility of Li_2_O_2_ formation–decomposition, leading to improved cycling stability [53].

Taken together, our findings provide compelling theoretical evidence correlating the enhanced performance of R-Co_3_O_4_/CP with the spin-polarized charge transport induced by the CISS effect. The integration of chiral molecular structures in R-Co_3_O_4_/CP enables electronic and spin modifications that significantly enhance catalytic efficiency, lower overpotentials, and suppress detrimental singlet oxygen formation. The experimental validation across electrochemical, spectroscopic, and titration-based characterizations strongly supports this connection, establishing a direct correlation between the CISS effect, charge transport modulation, and the suppression of ^1^O_2_. These results establish a direct correlation between the CISS effect, charge transport modulation, and the suppression of ^1^O_2_, offering a new design principle for high-performance Li–O_2_ batteries.

## Conclusions

Our work demonstrates that integrating the CISS effect into Li–O_2_ battery cathodes significantly enhances cell performance and longevity. Chiral Co_3_O_4_ nanosheets, electrodeposited using BINOL as a chirality inducer, exhibit improved oxygen electrochemistry, enhanced ORR and OER activities, and a substantial reduction in parasitic side reactions. *Operando* spectroscopic techniques, including DEMS and photoluminescence analysis, confirm effective suppression of singlet oxygen generation, a critical factor in mitigating cycling degradation. Complementary DFT calculations reveal that the introduction of chirality (via R-BINOL modification) alters the electronic structure of Co_3_O_4_, leading to a shift in the density of states near the Fermi level and enhanced orbital hybridization between Co 3*d* and O 2*p* states. These modifications contribute to lower overpotentials for both ORR and OER, promoting more efficient charge transfer. Additionally, adsorption energy calculations suggest that chiral Co_3_O_4_ favors a reaction pathway that stabilizes Li_2_O_2_ while weakening LiO_2_ adsorption, potentially influencing the deposition process. Collectively, our findings provide compelling evidence that chirality-driven electronic and spin modifications offer an effective strategy to regulate reaction pathways in Li–O_2_ batteries, suppress singlet oxygen generation, and enhance overall electrochemical stability. This pioneering approach opens promising avenues to integrate rational chiral material engineering toward the development of sustainable Li–O_2_ technologies with extended cycle life.

## Supplementary Information

Below is the link to the electronic supplementary material.

Supplementary file1 (DOCX 3794 KB)
