# Supplementary material for: Chirality-Induced Suppression of Singlet Oxygen in Lithium–Oxygen Batteries with Extended Cycle Life
Source: Nanomicro Lett. 2025 Aug 25;18:40. doi: 10.1007/s40820-025-01885-z (PMC12378250; doi:10.1007/s40820-025-01885-z)
Supplement: Supplementary file 1 — Supplementary file1 (DOCX 3794 KB) [file 40820_2025_1885_MOESM1_ESM.docx]

Supporting Information for

**Chirality-Induced Suppression of Singlet Oxygen in Lithium–Oxygen Batteries with Extended Cycle Life**

Kyunghee Chae,^1^ Youngbi Kim,^2^ Yookyeong Oh,^1^ Hosik Hahn,^3^ Jaehyun Son,^4^ Youngsin Kim,^3^ Hyuk-Joon Kim,^3^ Hyun Jeong Lee,^1^ Dohyub Jang,^5^ Jooho Moon,^4,^* Kisuk Kang,^3,^* Jeong Woo Han,^3,^* Filipe Marques Mota,^6,^* Dong Ha Kim^1,7,8,9,10,^*

^1^Department of Chemistry and Nanoscience, Ewha Womans University, 52 Ewhayeodae-gil, Seodaemun-gu, Seoul 03760, Republic of Korea

^2^Department of Chemical Engineering, Pohang University of Science and Technology (POSTECH), Pohang 37673, Republic of Korea

^3^Department of Materials Science and Engineering Seoul National University, 1 Gwanak-ro, Gwanak-gu, Seoul 08826, Republic of Korea

^4^Department of Materials Science and Engineering, Yonsei University, 50 Yonsei-ro Seodaemun-gu, Seoul 03722, Republic of Korea

^5^Chemical and Biological Integrative Research Center, Korea Institute of Science and Technology (KIST), 5 Hwarang-ro 14-gil, Seongbuk-gu, Seoul 02792, Republic of Korea

^6^Department of Chemistry, School of Natural Sciences, University of Lincoln, Brayford Pool, Lincoln LN6 7TS, United Kingdom

^7^College of Medicine, Ewha Womans University, 25, Magokdong-ro 2-gil, Gangseo-gu, Seoul 07804, Republic of Korea

^8^Gradutate Program in Innovative Biomaterials Convergence, 52 Ewhayeodae-gil, Seodaemun-gu, Ewha Womans University, Seoul 03760, Republic of Korea

^9^Basic Sciences Research Institute (Priority Research Institute), Ewha Womans University, Seoul 03760, Republic of Korea

^10^Nanobio • Energy Materials Center (National Research Facilities and Equipment Center), Ewha Womans University, Seoul 03760, Republic of Korea

*Corresponding authors. E-mail: [jmoon@yonsei.ac.kr](mailto:jmoon@yonsei.ac.kr) (Jooho Moon); [matlgen1@snu.ac.kr](mailto:matlgen1@snu.ac.kr) (Kisuk Kang); [jwhan98@snu.ac.kr](mailto:jwhan98@snu.ac.kr) (Jeong Woo Han); [FMarquesMota@lincoln.ac.uk](mailto:FMarquesMota@lincoln.ac.uk) (Filipe Marques Mota); [dhkim@ewha.ac.kr](mailto:dhkim@ewha.ac.kr) (Dong Ha Kim)

**S1 Supplementary Method**

**S1. 1 Electrochemical linear sweep voltammetry**

Linear sweep voltammetry (LSV) tests of Co_3_O_4_/CP electrodes were performed using typical three-electrode system with an electrochemical workstation (SP150) at room temperature. A graphite rod and Ag/AgCl were used as the counter and the reference electrodes, respectively. The described setup was used to investigate both OER and ORR profiles in 1 M KOH aqueous solution at a scan rate of 20 mV s^-1^, with as-prepared electrodes in each N_2_- and O_2_-saturated electrolyte.

**S1.2 Acid-based titration**

Inside the Ar-filled glove box, spent cathodes along with the soaked pieces of Celgard, microfiber separators, and stainless-steel meshes were directly placed into vials containing 5 mL of deionized water and gently shaken for a few minutes. A complete dissolution and reaction of present DC products with H_2_O were assumed as Li_2_O_2_ + 2H_2_O → H_2_O_2_ + 2LiOH. During the acid-base titration process to examine the formation of Li_2_O_2_, possible side reactions with atmospheric CO_2_ could lead to the formation of Li_2_CO_3_, potentially affecting the accuracy of the results. To minimize this effect, all titrations were conducted inside an Ar-filled glovebox to prevent CO_2_ exposure. The extracted solution was titrated immediately (within 5 minutes of Li_2_O_2_ extraction) using a freshly prepared 5 mM HCl aqueous solution as the titrant (LiOH + HCl = LiCl + H_2_O). Titration was performed dropwise under gentle stirring until the phenolphthalein indicator (20 μL, 1% in EtOH) changed color, indicating the neutralization of OH^-^ species. To ensure reproducibility, at least three separate titrations were conducted for each sample, and the average Li_2_O_2_ yield was reported with standard deviation. Calibration was confirmed using a known LiOH standard (Fig. S9), and blank control titration were performed with fresh electrolyte-exposed separators and cathodes without cycling to confirm the absence of background alkalinity contributions.

**S1.3 UV-vis analysis**

UV-vis absorption spectra were recorded with a UV–Vis Varion 5000 spectrometer with a wavelength rate of 300-500 nm. TEGDME electrolytes containing 20 mM DMA were used as the original solution. After discharge and recharge for 1 hour at 0.08 mA cm^-2^, spent cathodes along with the soaked pieces of Celgard, microfiber separators, and stainless-steel meshes were diluted with TEGDME. Then, UV absorption spectra were recorded.

**S2 Supplementary Figures and Tables**


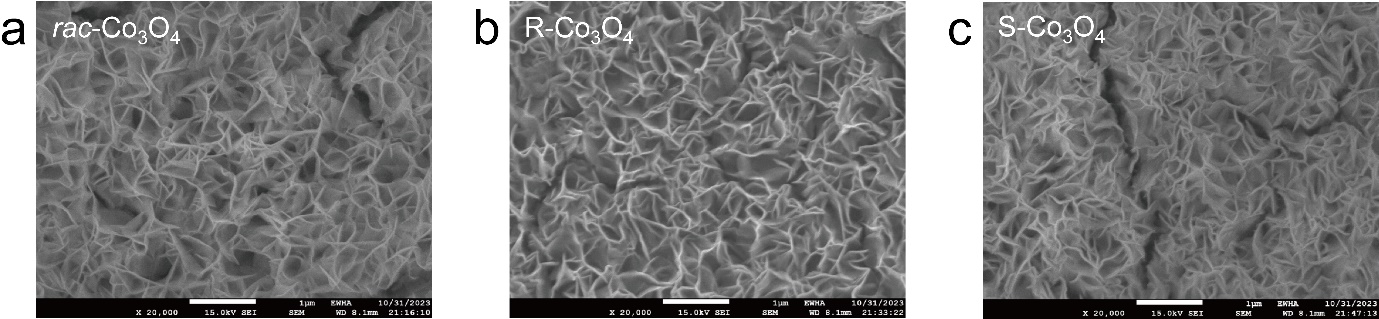


**Fig. S1** SEM images of (**a**) *rac*-Co_3_O_4_ NS/ITO, (**b**) R-Co_3_O_4_ NS/ITO and (**c**) S-Co_3_O_4_ NS/ITO substrates


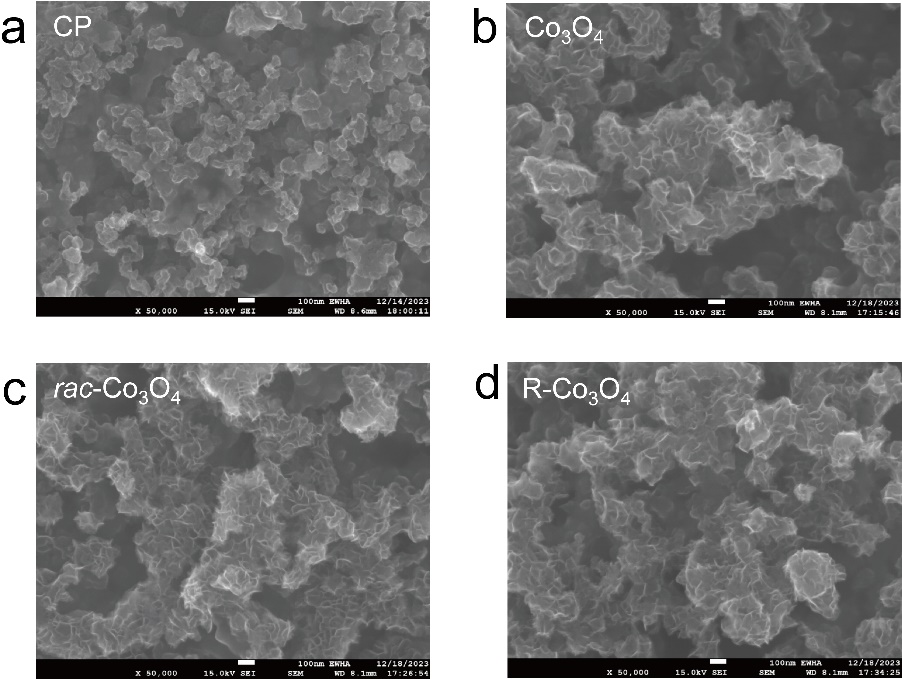


**Fig. S2** SEM images of (**a**) bare carbon paper (CP), (**b**) Co_3_O_4_ NS/CP, (**c**) *rac*-Co_3_O_4_ NS/CP, and (**d**) R-Co_3_O_4_ NS/CP


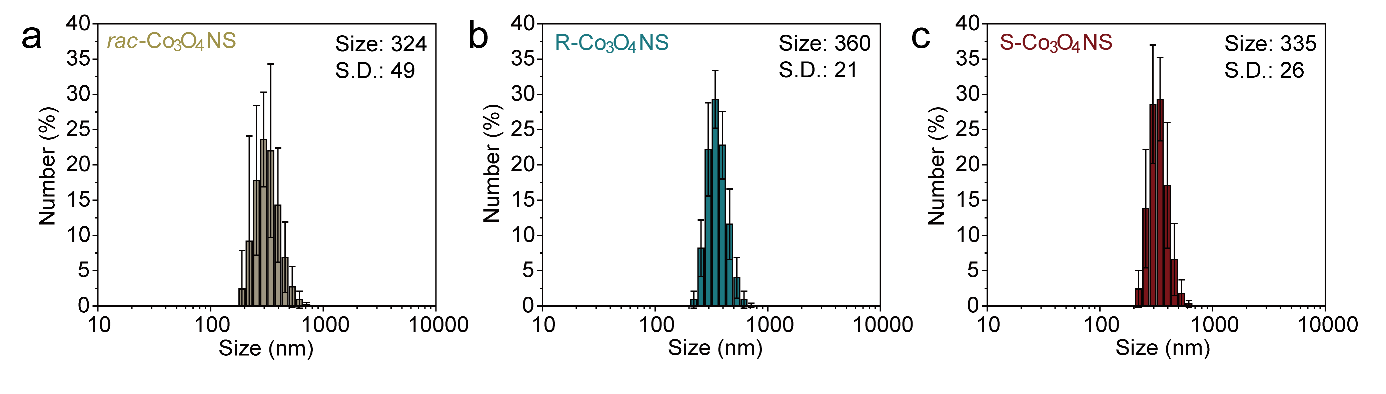


**Fig. S3** Dynamic light scanning (DLS) size distribution of (**a**) *rac*-Co_3_O_4_ NS, (**b**) R-Co_3_O_4_ NS, and (**c**) S-Co_3_O_4_ NS


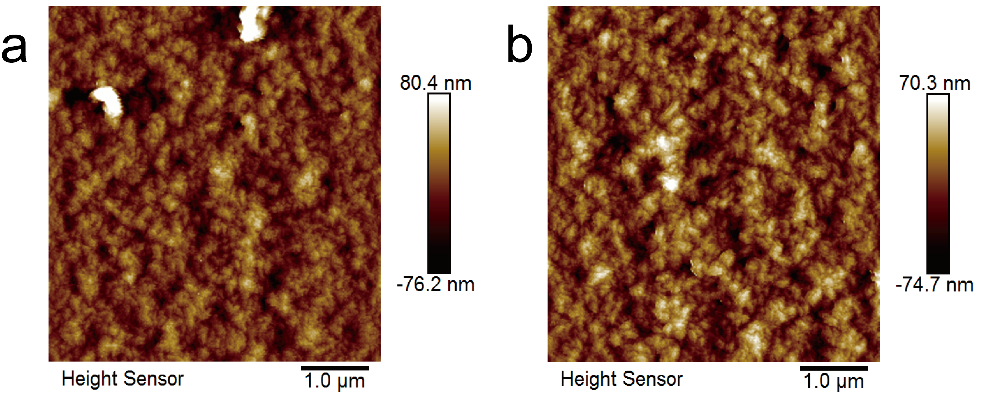


**Fig. S4** AFM height images of electrodeposited Co_3_O_4_ nanosheets on ITO substrates. (**a**) *rac*-Co_3_O_4_/CP and (**b**) R-Co_3_O_4_/CP


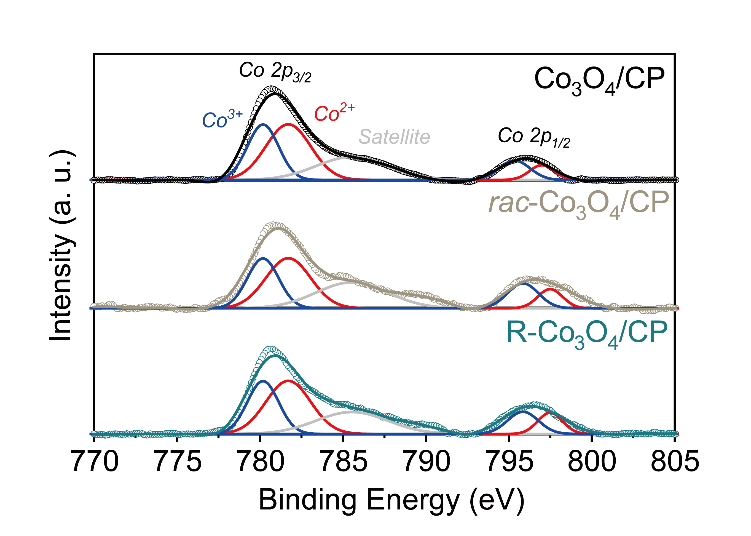


**Fig. S5** XPS spectra of Co_3_O_4_ NS/CP, *rac*-Co_3_O_4_ NS/CP and R-Co_3_O_4_ NS/CP. All samples show deconvoluted Co^2+^ and Co^3+^ peaks along with their satellite features, confirming the typical mixed-valence nature of spinel Co_3_O_4_. The calculated Co^2+^/Co^3+^ ratios were 1.44 for Co_3_O_4_/CP, 1.46 for *rac*-Co_3_O_4_/CP, and 1.43 for R-Co_3_O_4_/CP, indicating consistent chemical states across samples


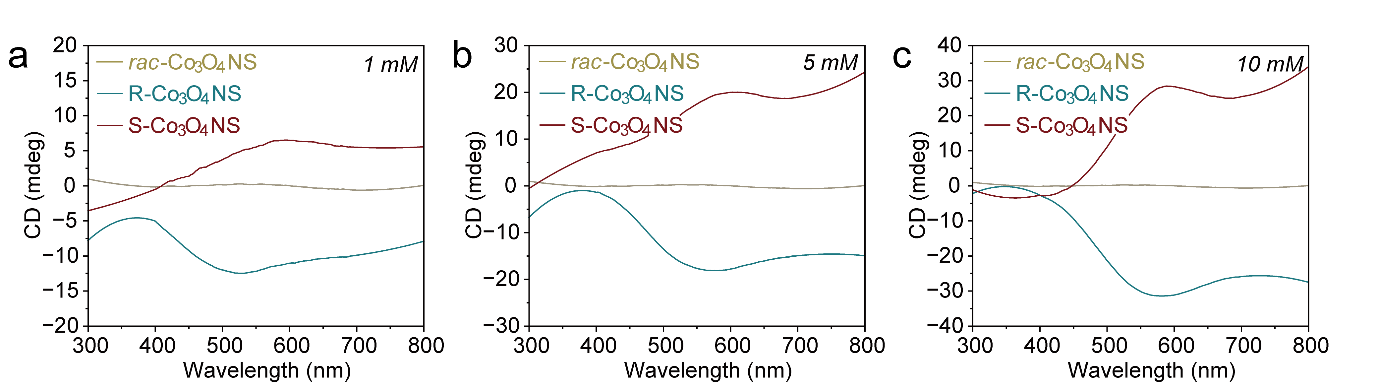


**Fig. S6** CD spectra of (*rac*/R/S)-Co_3_O_4_ NS on an ITO substrate with (**a**) 1 mM, (**b**) 5 mM, and (**c**) 10 mM of (*rac*/R/S)-BINOL


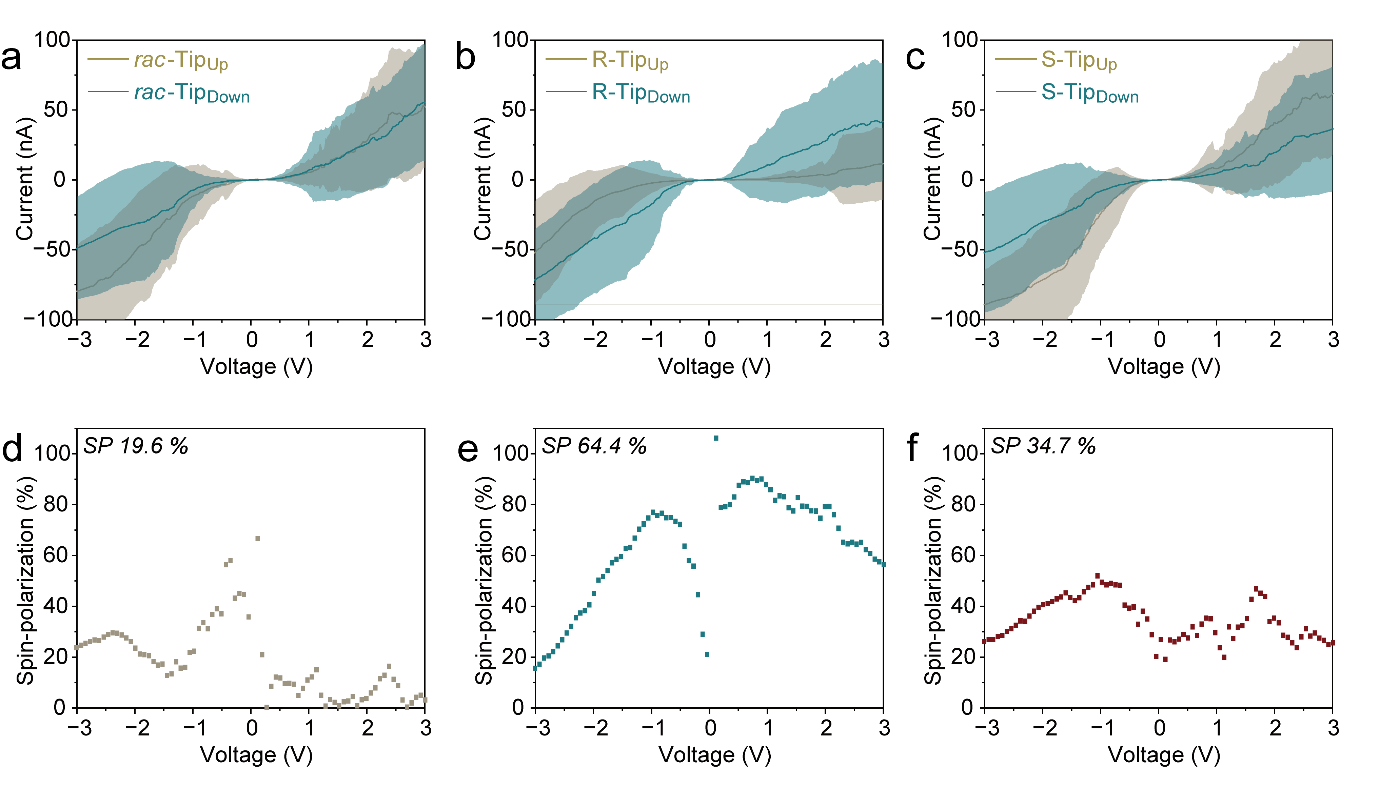


**Fig. S7** *I-V* curves of (**a**) *rac*-Co_3_O_4_/CP, (**b**) R-Co_3_O_4_/CP and (**c**) S-Co_3_O_4_/CP electrodes in the range of -3.0 to +3.0 V. The CoCr tip was magnetized along the north (gray) or south (green) orientation. The average *I-V* curve recorded over 30 scans at different points is shown. Spin polarization percentage (SP%) as a function of applied bias of (**d**) *rac*-Co_3_O_4_/CP, (**e**) R-Co_3_O_4_/CP and (**f**) S-Co_3_O_4_/CP electrodes


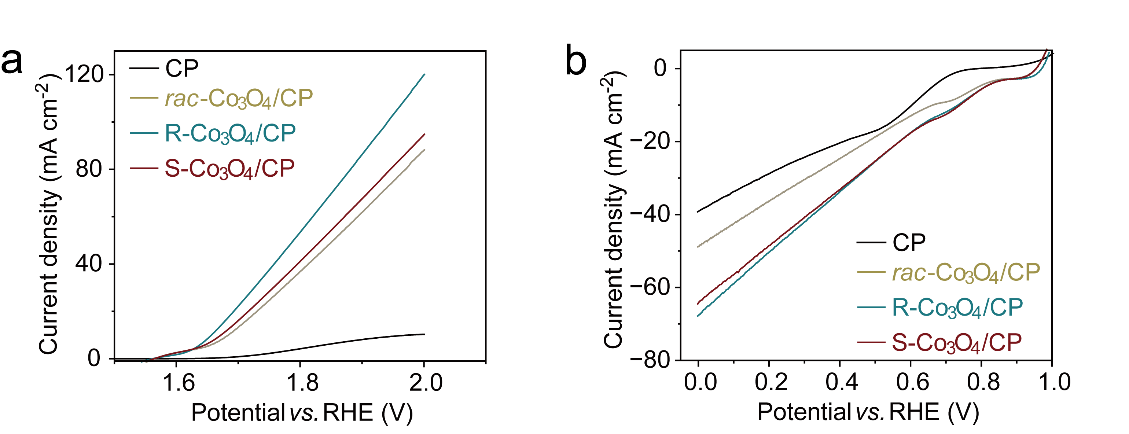


**Fig. S8** (**a**) OER LSV curves of CP and (*rac*/R/S)-Co_3_O_4_/CP electrodes in the N_2_-saturated 1 M KOH electrolyte. (**b**) ORR LSV curves of CP and (*rac*/R/S)-Co_3_O_4_/CP electrodes in O_2_-saturated 1 M KOH electrolyte (subtracting the voltammograms in N_2_). Scan rate is 20 mV s^-1^


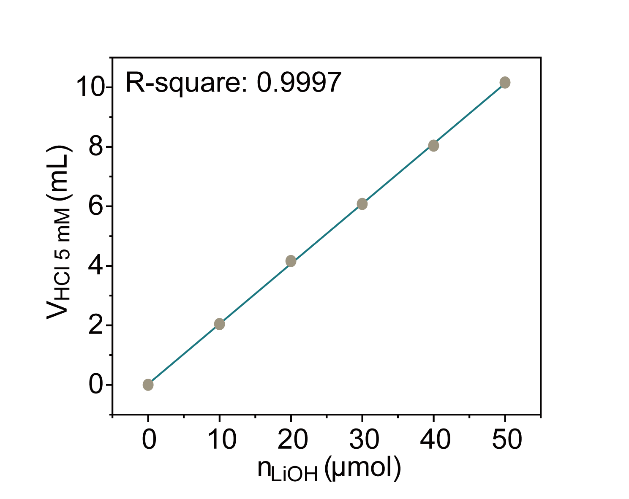


**Fig. S9** Acid-base titration calibration curve with phenolphthalein indicator

To quantify the Li_2_O_2_ formed during the first discharge process, we conducted acid-base titration experiments following a 5-hour discharge at a current density of 0.8 mA cm^–2^, with a total capacity of 4 mAh/cm^2^. This titration process is based on the reaction of lithium peroxide (Li_2_O_2_) with water, which produces lithium hydroxide (LiOH), as shown in the reaction:

Li_2_O_2_ + 2H_2_O → H_2_O_2_ + 2LiOH

Here, the relationship between LiOH and Li_2_O_2_ follows a 2:1 molar ratio. The resulting LiOH can then be titrated using a standardized hydrochloric acid (HCl) solution, which reacts with LiOH to form lithium chloride (LiCl) and water.

For theoretical calculations, based on a 5-hour discharge at a capacity of 0.4 mAh cm^–2^ and an electrode surface area of 1.25668 cm^2^, the total discharge capacity is 0.5067 mAh (or 0.000507 Ah). This corresponds to 18.9 µmol of LiOH, given the two-electron transfer required for Li_2_O_2_ formation. Therefore, after 5 hours of discharge, the theoretical amount of Li_2_O_2_ produced should be 9.45 µmol, generating 18.9 µmol of LiOH in the titration process.


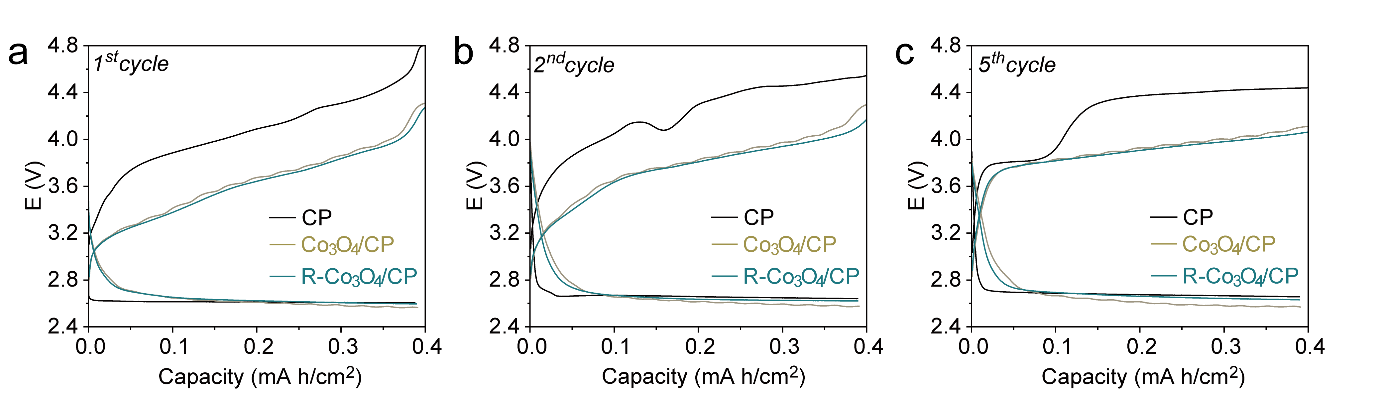


**Fig. S10** Galvanostatic profiles of Li–O_2_ batteries with CP, Co_3_O_4_ NS/CP and R-Co_3_O_4_ NS/CP at 0.08 mA cm^-2^ for (**a**) 1^st^ cycle, (**b**) 2^nd^ cycle and (**c**) 5^th^ cycle


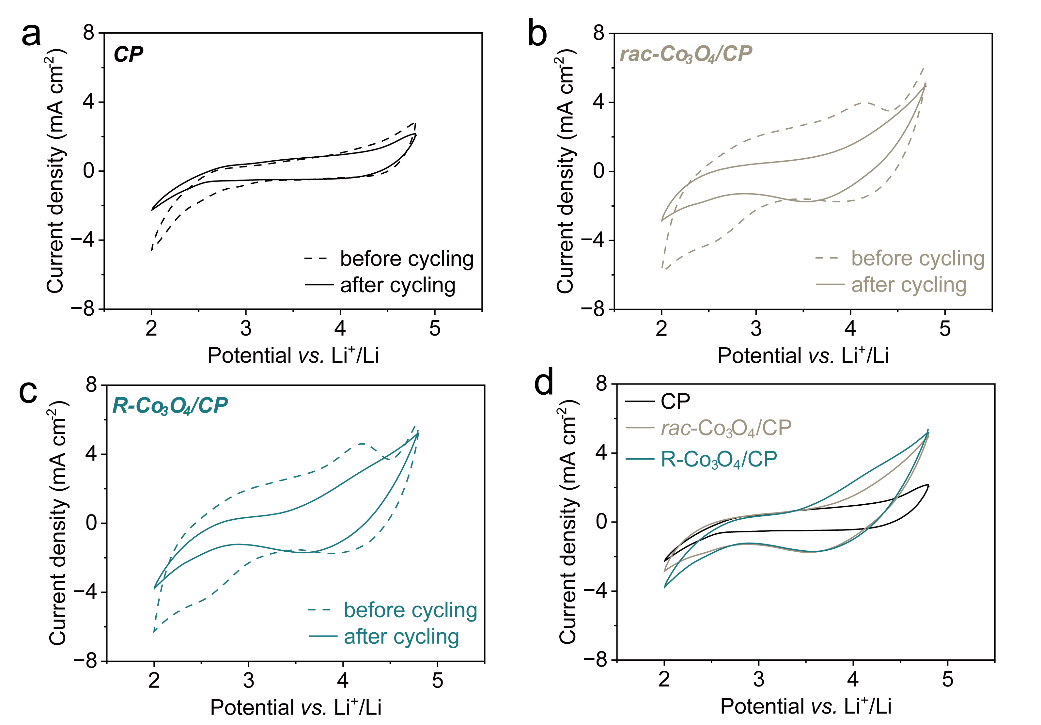


**Fig. S11** Cyclic voltammetry (CV) curves of (**a**) CP, (**b**) *rac*-Co_3_O_4_/CP, and (**c**) R-Co_3_O_4_/CP electrodes measured before and after 10 discharge/recharge galvanostatic cycles at a scan rate of 0.1 mV s^-1^ in the voltage range of 2.0-4.8 V vs. Li^+^/Li. The 10-cycle test was conducted at a current density of 0.08 mA cm^-2^ using a fixed charge/discharge time of 10 hours per cycle. (**d**) Overlaid CV profiles after cycling, demonstrating the superior catalytic stability of R-Co_3_O_4_/CP, which retains higher ORR and OER current densities compared to *rac*-Co_3_O_4_/CP and CP, indicating enhanced durability under prolonged cycling


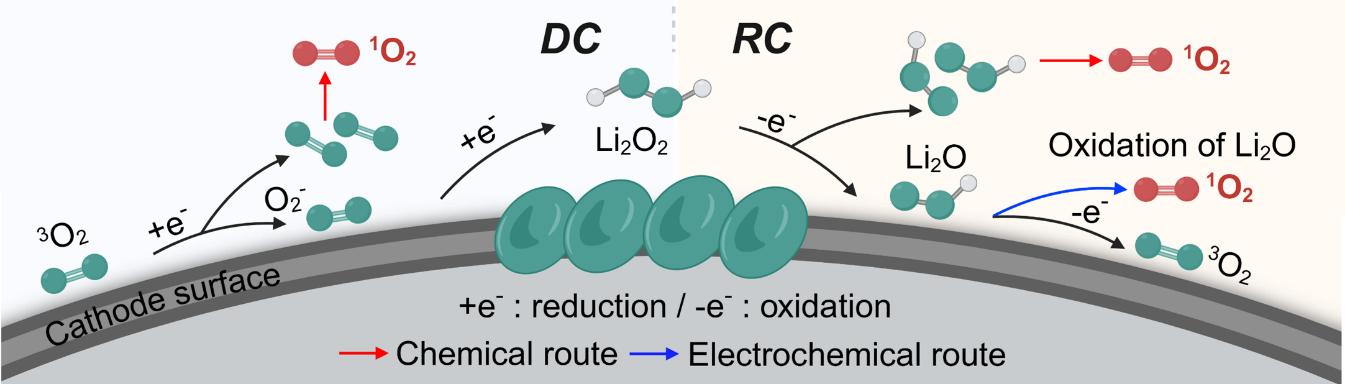


**Fig. S12** Schematic generation of ^1^O_2_ during discharge and charge processes


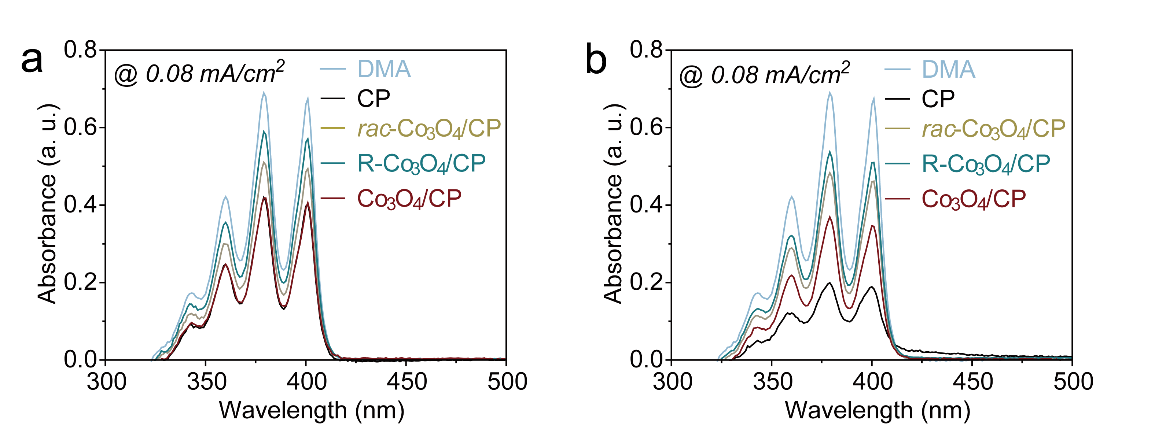


**Fig. S13** UV-vis absorption spectrum of DMA in TEGDME with CP, Co_3_O_4_/CP and (*rac*/R)-Co_3_O_4_/CP electrodes after discharge (**a**) and recharge (**b**) for 1 hour at 0.08 mA cm^-2^


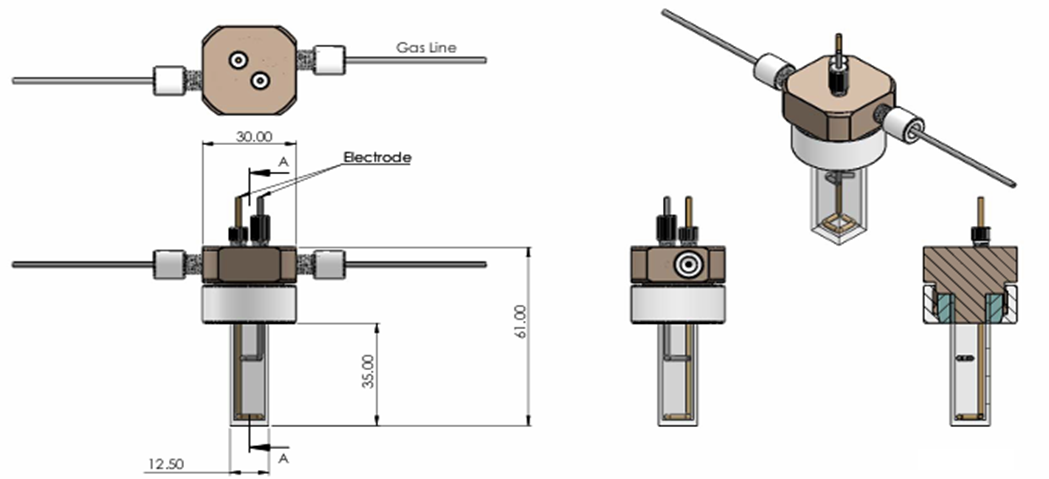


**Fig. S14** Schematic diagram of operando PL cell design


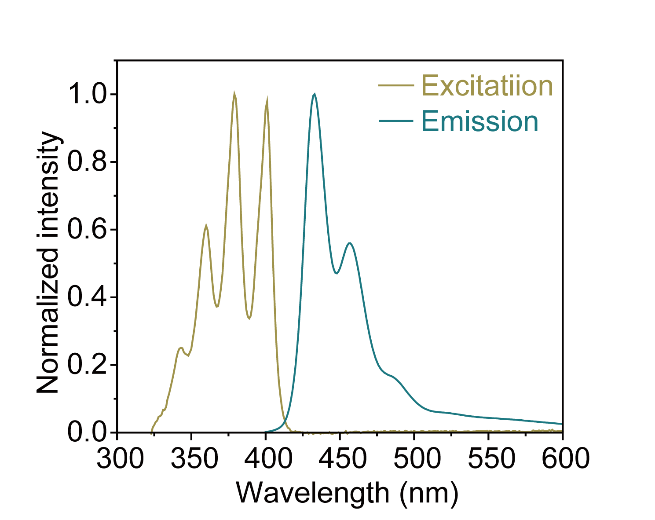


**Fig. S15** Excitation and emission spectra of 9,10-dimethylanthracene in 1.0 M LiTFSI in TEGDME. The emission was recorded from 400-600 nm with λ_ex_= 378nm


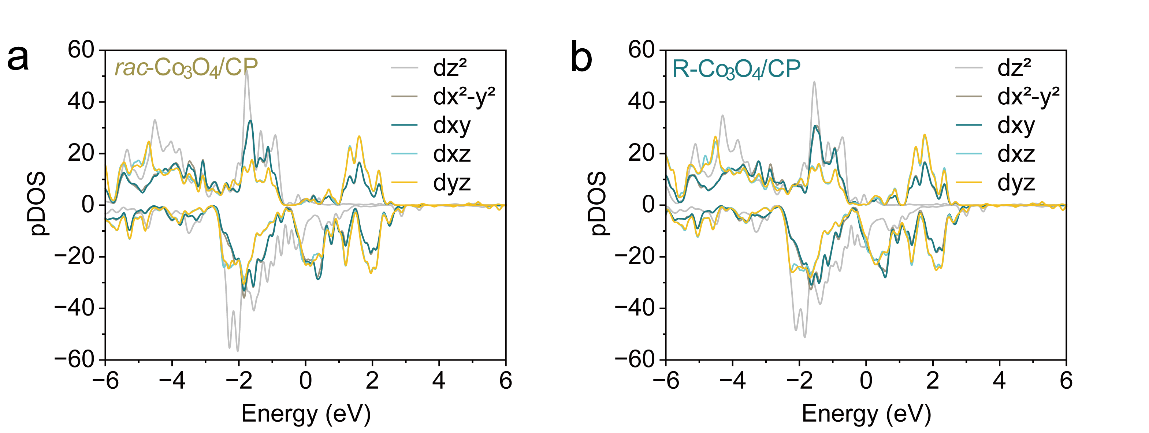


**Fig. S16** Partial density of states (PDOS) for Co 3d orbitals in (**a**) *rac*-Co_3_O_4_ NS/CP and (**b**) R-Co_3_O_4_ NS/CP


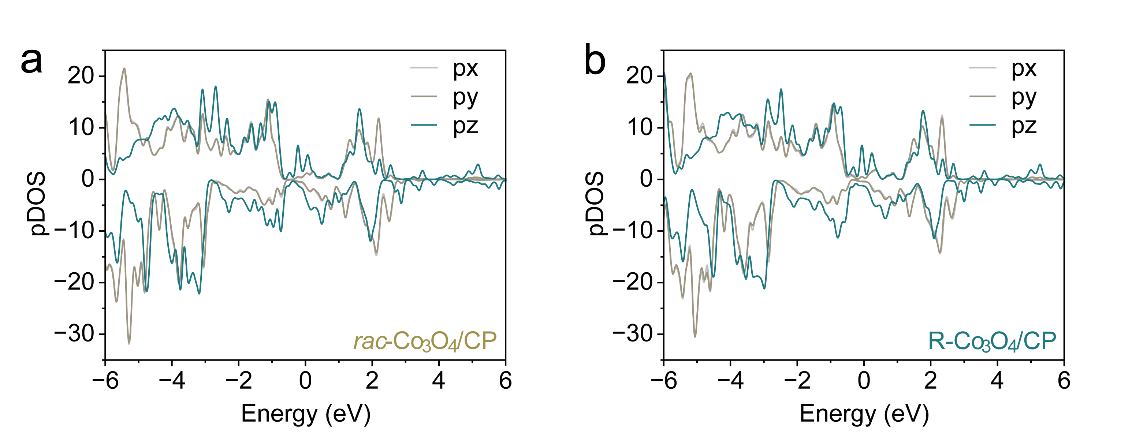


**Fig. S17** Partial density of states (PDOS) for O 2p orbitals in (**a**) *rac*-Co_3_O_4_ NS/CP and (**b**) R-Co_3_O_4_ NS/CP


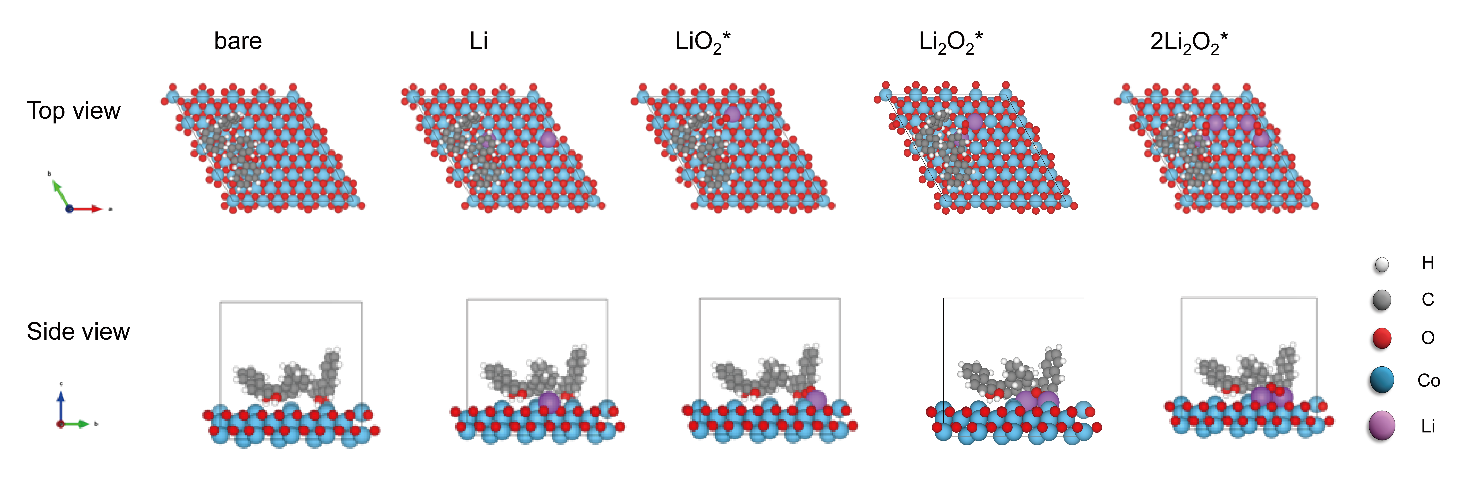


**Fig. S18** The computed geometries of bare, Li, LiO_2_*, Li_2_O_2_*, and 2Li_2_O_2_* at the *rac*-Co_3_O_4_ NS/CP cathode


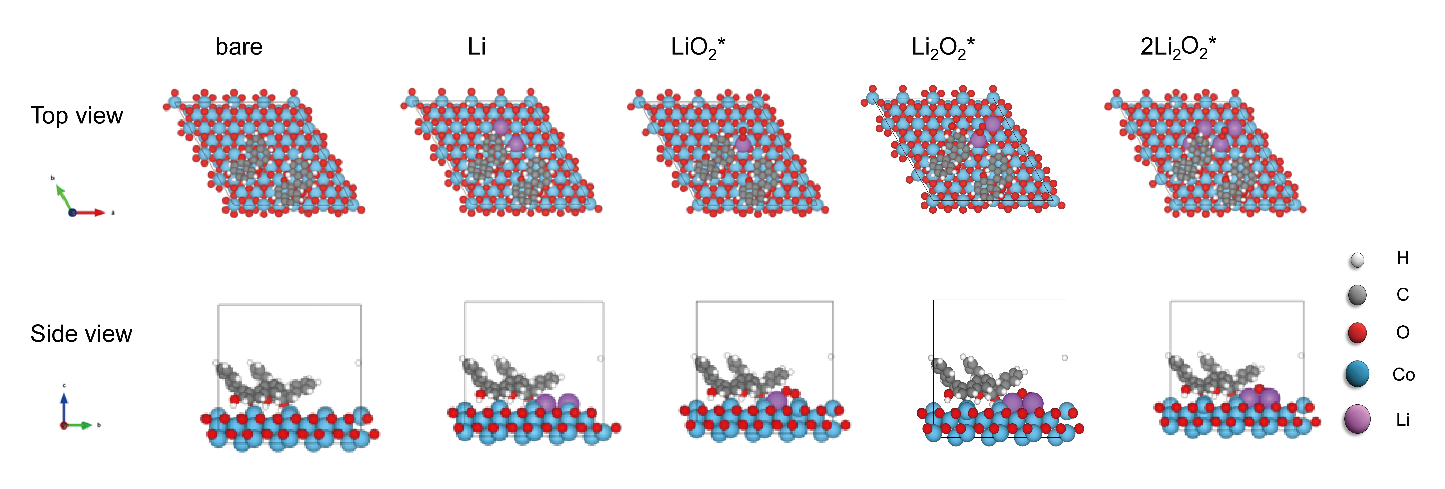


**Fig. S19** The computed geometries of bare, Li, LiO_2_*, Li_2_O_2_*, and 2Li_2_O_2_* at the R-Co_3_O_4_ NS/CP cathode

**Table S1** Summary of ICP-OES results and relative standard deviation values (RSD, %)

| **Sample name** | **Co 238.892 nm** | | **Sample weight (g)** |
| --- | --- | --- | --- |
|  | **Conc. (wt%)** | **RSD** |  |
| *rac*-Co_3_O_4_/CP | 5.274 | 1.579 | 0.0128 |
| R-Co_3_O_4_/CP | 5.006 | 2.190 | 0.0138 |
| S-Co_3_O_4_/CP | 6.149 | 0.797 | 0.0133 |
